# Estimation of the Underlying Burden of Pertussis in Adolescents and Adults in Southern Ontario, Canada

**DOI:** 10.1371/journal.pone.0083850

**Published:** 2013-12-23

**Authors:** Ashleigh A. McGirr, Ashleigh R. Tuite, David N. Fisman

**Affiliations:** Division of Epidemiology, Dalla Lana School of Public Health, University of Toronto, Toronto, Ontario, Canada; Arizona State University, United States of America

## Abstract

Despite highly successful vaccination programs and high vaccine uptake, both endemic pertussis and periodic pertussis outbreaks continue to occur. The under-recognized role of adolescents and adults in disease transmission, due to waning immunity following natural infection and vaccination, and reduced likelihood of correct diagnosis, may contribute to pertussis persistence. We constructed a mathematical model to describe the transmission of pertussis in Southern Ontario in both pre-vaccine and vaccine eras, to estimate the underlying burden of pertussis in the population. The model was well calibrated using the best available data on pertussis in the pre-vaccination (1880–1929) and vaccination (1993–2004) eras in Ontario. Pertussis under-reporting by age group was estimated by comparing model-projected incidence to reported laboratory-confirmed cases for Greater Toronto. Best-fit model estimates gave a basic reproductive number of approximately 10.6, (seasonal range 9.9 to 11.5). Under-reporting increased with age, and approximately >95% of infections in children were caused by infections in persons with waning immunity to pertussis following prior infection or vaccination. A well-calibrated model suggests that under-recognized cases of pertussis in older individuals are likely to be an important driver of ongoing pertussis outbreaks in children. Model projections strongly support enhancement of booster vaccination efforts in adults.

## Introduction

Pertussis is a highly contagious respiratory tract infection caused by the gram negative bacterium *Bordetella pertussis*, or less commonly by *B. parapertussis* [1]. While children and adults of any age may develop pertussis, severe sequelae (including encephalopathy and pneumonia) are most common in young infants [2], [3], [4]. The disease remains one of the leading causes of infant mortality, causing 300,000 deaths and 50 million cases per year, mostly in countries lacking the resources to support widespread immunization [3], [5].

With introduction of pertussis immunization in Canada in the 1940s, annual pertussis incidence decreased dramatically (from over 140 cases per 100,000 to fewer than 20 cases per 100,000 by the 1970s [6]). However, despite high levels of vaccine uptake in Canada, the disease has not been eliminated. Periodic pertussis outbreaks continue to present a challenge [7], [8], with recent large outbreaks or increases in pertussis incidence occurring in infants in high income countries including United States, Canada, Norway, Ireland, Australia and the United Kingdom [6], [7], [8], [9], [10], [11], though in middle income countries with longstanding vaccine programs, such as Thailand, resurgences have been absent [12]. Disease incidence also appears to be increasing [2], , a phenomenon variously attributed to changing vaccine preparations, aging of under-vaccinated cohorts, bacterial mutation, and more sensitive laboratory testing. [1], [3], [6], [13], [15], [16], [17], [18], [19], [20].

Another proposed explanation for the persistence of pertussis is the under-recognized role of adolescents and adults in disease transmission [21], due to waning immunity following natural infection and vaccination [3], [13], [22], and decreased likelihood of diagnosis, due to different disease manifestation in these groups, compared to that observed in infants and children [23]. Furthermore, widespread adoption of vaccination, but at a level insufficient to result in disease elimination, could eliminate natural “boosting” through interactions between previously infected individuals and infectious cases, further contributing to loss of immunity in older individuals [24], [25]. Indeed, a recent community-based study of cough illness performed in Poland suggested that pertussis in older adults might be under-reported by a factor of 167, in contrast to 4-fold under-reporting in children aged 3 to 5 [26]. Given the apparent importance of adolescents and adults in disease spread, several countries have advocated implementing a booster dose of pertussis vaccine [27], [28], though others have suggested that age-assortative mixing would mean that boosting in adolescents might have little impact on disease impact in infants [11].

To better understand how under-recognition of pertussis in adolescents and adults may contribute to observed disease patterns, we constructed a mathematical model to describe the pertussis transmission in the Canadian province of Ontario. We used this model to estimate the underlying burden of pertussis in the population, and to derive credible estimates of the likely degree of underdiagnosis of pertussis in older individuals that would be necessary to explain current observed epidemiological trends.

## Methods

### Pertussis Model Construction

**Figure 1:**
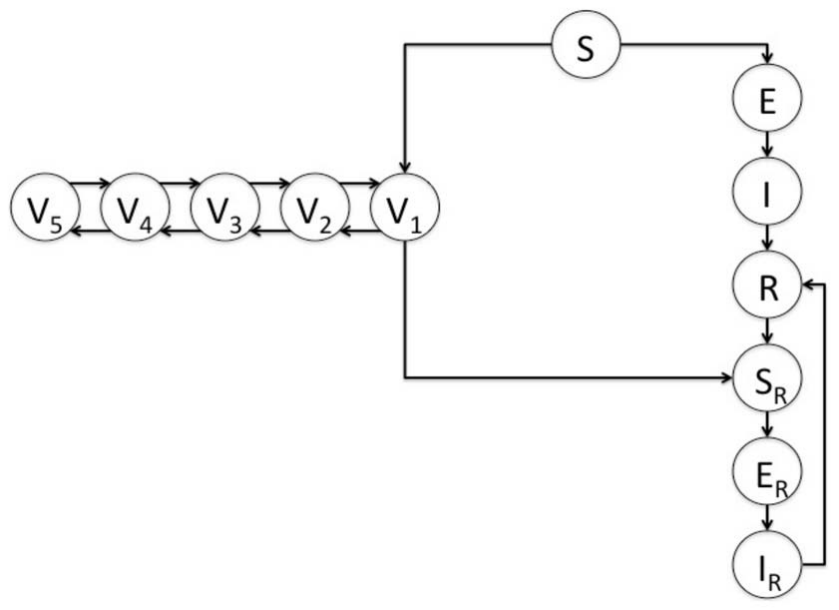
Model overview. The population was divided into eight different disease states: susceptible (S), vaccinated (V), exposed (E, infected but not infectious), infectious (I), recovered (R), re-susceptible (S_R_), re-exposed (E_R_), and re-infectious (I_R_). Each vaccination compartment (V_1_…V_5_) represents a different level of conferred immunity as children progress through the 5 recommended childhood pertussis vaccines. For complete model details refer to **File S1**.

We used Berkeley Madonna [29] to construct an age-structured compartmental model that included births and deaths, and the introduction of the pertussis vaccine, in order to examine multi-year pertussis dynamics (see **Model Description in File S1** for additional model details). The basic model structure is presented in detail in **Figure 1**. Natural history parameters (**Table 1**) were derived from epidemiologic studies and by model calibration. The population was divided into eight different disease states: susceptible (S), vaccinated (V), exposed (E, infected but not infectious), infectious (I), recovered (R), re-susceptible (S_R_), re-exposed (E_R_), and re-infectious (I_R_). Transmission of infection occurred through contact between susceptible or re-susceptible and infectious individuals. As individuals lost naturally-acquired or vaccine-induced immunity over time, they became re-susceptible to infection; we assumed that these individuals were equally susceptible as pertussis-naïve individuals, but were one-fifth as infectious (i.e., less likely to spread infection to others as assumed in previous models [30]). This decrease in infectiousness incorporates both the hypothetical vaccine efficacy for infectiousness [31] and the reduced duration of cough observed in partially immunized individuals [32].

**Table 1:** Parameter values used in model. Parameters used in the sensitivity analysis were drawn from uniform distributions with the plausible ranges indicated.

Parameter	Best-fit value (plausible range for sensitivity analysis)	Source
Latent period (days)	8	Nguyen and Rohani, 2008[35]
Infectious period (days)	15	Nguyen and Rohani, 2008[35]
Duration of immunity following infection (years)	17.84 (10–50)	Assumption; Wendelboe *et al*., 2005[41]; Wearing and Rohani, 2009[40]
Duration of immunity following complete immunization (years)	27.11 (2–30)	Model calibration; Wendelboe *et al.*, 2005[41]
Relative infectiousness of individuals re-challenged with pertussis (following loss of naturally-acquired or vaccine-induced immunity)	0.2	Assumption, similar to van Boven *et al*., 2000 [30]
β_1_, base transmission parameter	11.33 (9–12)	Model calibration
β_2_, relative amplitude of annual forcing	0.05 (0.005–0.1)	Model calibration
β_3_, relative amplitude of seasonal forcing	0.029 (0.01–0.05)	Model calibration
Life expectancy pre-vaccine era (years)	66	Assumption
Life expectancy in vaccine era (years)	75	Assumption
Vaccine Efficacy	0.9	Schmitt *et al.*, 1996 [52], Preziosi and Halloran, 2003 [31], Ward *et al.*, 2005 [53]

To model existing pertussis vaccination schedules and to enable the representation of more realistic contact patterns within and between age groups, our model was age-structured, with ten age classes based in part on vaccine recommendations (<2 months, 2–4 months, 4–6 months, 6 months–2 years, 2–7 years, 7–10 years, 10–15 years, 15–20 years, 20–65 years and 65+ years). Mixing within and between age strata was based on the best available survey data for high-income countries [33]. The original survey was conducted in Great Britain with a nationally representative sample of participants who maintained diaries of the age and gender of all contacts with which they had a two-way conversation consisting of more than 3 words per day. The results from Great Britain echoed similar findings from Belgium, Germany, Finland, Italy, Luxembourg, The Netherlands, and Poland in terms of the age-specific contact patterns, suggesting that this contact matrix is robust. The contact matrix used in the present analysis was modified from [33] to capture the age categories specific to the ten age classes used in the model. The full contact matrix can be found in **File S1.**

The birth rate was set equal to the death rate to maintain a constant population size and population distribution among age classes. Deaths occurred only in the oldest age group, with continuous aging through the age cohorts. Loss of immunity following immunization or natural infection was incorporated using existing estimates and model calibration. While the vaccine preparation offered in Ontario changed from adsorbed whole cell vaccine to an acellular preparation in 1997–98, the two preparations exhibited similar levels of efficacy so were treated as such in the model [34]. Latent and infectious periods were assumed to follow a gamma distribution [35]. Additional information on model parameter values is presented in **Table 1**.

Periodicity of pertussis epidemics was simulated by forcing the effective contact rate (*β*) to oscillate through time. This was done by incorporating two cosine terms into the base model transmission parameter (*β_1_*) to represent annual outbreaks (*β*_2_) and epidemics every 4 years (*β*_3_), such that:
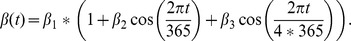



Vaccination was modeled as a continuous process whereby individuals were moved into different vaccination compartments as they entered the different age classes at which pertussis vaccine is typically administered (i.e., as they enter the 2 month, 4 month, 6 month, 2 years, or 7 years of age categories), resulting in a total of 5 vaccinated compartments. Upon administration of the first dose at 2 months, a susceptible individual was moved into the V_1_ compartment, and with each subsequent dose moved up to the next vaccination compartment (V_2_ to V_5_). The final distribution of individuals among the 5 vaccination compartments in the 7–10 age category (i.e., after administration of the final possible vaccine dose) was specifically designed using age-specific probabilities of vaccination to reflect existing Canadian pertussis vaccine coverage estimates (**Table 2**) [36]. We assumed that the individuals in the vaccinated compartments were fully protected against pertussis infection, with the remaining fraction receiving no protection. Receipt of each vaccine dose was assumed to boost immunity to infection, and with loss of vaccine-induced immunity, an individual was moved to a lower vaccine compartment and ultimately returned to the re-susceptible class (i.e., from V_k_ to V_k-1_ or V_1_ to S_R_).

**Table 2:** Estimated pertussis vaccine coverage at 7 years of age.

Number of doses	Recommended age at vaccination	Reported (PHAC)^a^	Model
0		0.04	0.034
1	2 months	0.02	0.022
2	4 months	0.04	0.043
3	6 months	0.06	0.067
4	1.5 years	0.19	0.183
5	4–6 years	0.65	0.652

^a^ McWha L, MacArthur A, Badiani T, Schouten H, Tam T, et al. (2004) Measuring up: results from the National Immunization Coverage Survey, 2002. Can Commun Dis Rep 30: 37–50.

### Model Calibration

In order to credibly calibrate our model, we used a two-step procedure, first calibrating pertussis incidence in an unvaccinated population to the best available data on pre-vaccination pertussis incidence in Ontario (1880–1929), and subsequently calibrating vaccine effectiveness and durability estimates using more recent time series data (1993–2004). Specifically, we used reported proportionate mortality by age group [37] and applied age-specific case-fatality ratios (estimated in 32 U.S. cities over a ten-year period) [38] to calculate expected pertussis incidence between 1880 and 1929. The time series data for pertussis incidence in the vaccine-era was obtained from the two pertussis testing laboratories in the Greater Toronto Area (GTA): the Public Health Laboratory- Toronto (PHLT) and the Hospital for Sick Children (HSC). This dataset contains laboratory confirmed pertussis cases (by culture and PCR) from 1993 to 2004. Calibration was performed using Berkeley Madonna modeling software, which determines the best fitting estimates based on minimizing the root mean square deviation between the dataset and the predicted outputs from each run [29], [39]. Full details on the model calibration procedure can be found in **File S1.**

### Model Validation

In order to validate the model, the secondary model outputs including the estimated reproductive number and duration of vaccine-induced immunity were compared with previously cited literature values.

### Estimation of Pertussis Under-identification

To determine the degree of pertussis under-identification, we compared model projected age-specific annual incidence to the 1993–2004 time series. The ratio of projected to reported cases was estimated using the mean daily cumulative incidence over this time period. The contribution of infection in persons previously exposed to pertussis, either through natural infection or vaccination, to the force of infection (the rate at which susceptible individuals become infected), was calculated as:
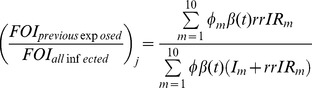
where *j* is the age group of the susceptible population, *m* is the age group of the infectious contacts, *I_m_* is the number of previously pertussis-naïve infectious individuals of age *m*, *IR_m_* is the number of previously exposed or vaccinated infectious individuals of age group *m*, *rr* is the relative infectiousness of previously exposed individuals, and *φ_j,m_β*(t) is the probability of effective infectious contact between an infectious and susceptible individual.

### Estimation of Vaccine-Induced Immunity Among Older Individuals

In order to estimate the proportion of individuals in each age group with vaccine-induced immunity to pertussis, we calculated the model predicted distribution of individuals in each of the vaccination compartments, V_1_ to V_5_, for each of the age groups at the end of the 2012 calendar year. We then used the derived probabilities of vaccination for each age group to calculate the estimated proportion of individuals who had received at least one pertussis vaccination within each of the age groups.

### Sensitivity Analyses

Given the uncertainty around parameters describing the natural history of pertussis, we conducted a multi-way sensitivity analysis. Parameters were drawn from uniform distributions, with the ranges outlined in **Table 1**, for 1000 simulations. Age-specific probabilities of reported diagnosis were calculated for each model run and summarized with parametric confidence limits.

## Results

### Calibration

**Figure 2:**
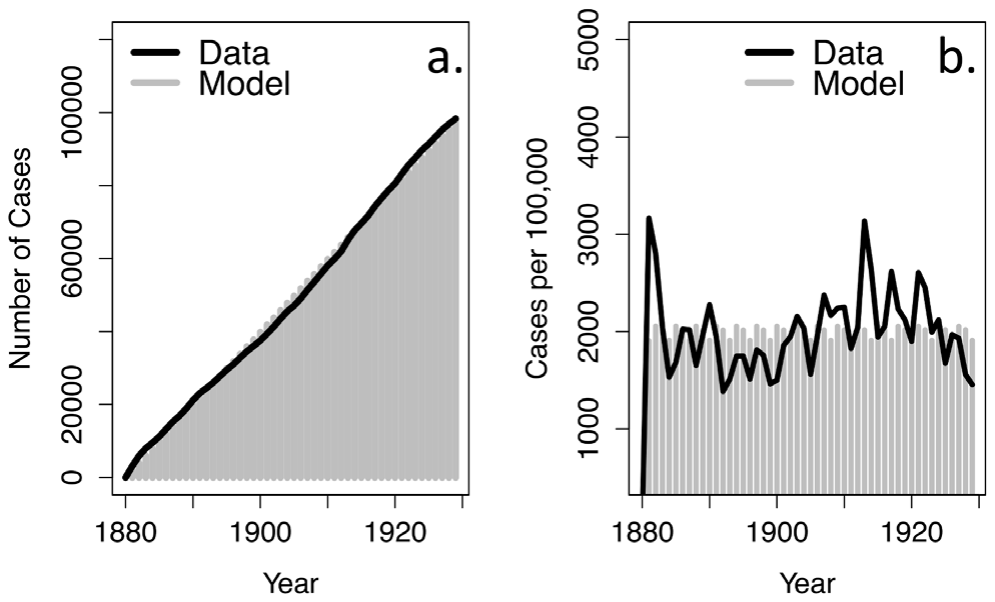
Model calibration to pertussis incidence in the pre-vaccine era. Model-projected (a) cumulative pertussis incidence and (b) annual pertussis incidence in the population under 2 years old (bars) were fit to a time-series of pertussis incidence between 1880 and 1929 (line). Best-fit parameter estimates were: *β_1_* of 11.335, *β_2_* of 0.050, and *β*_3_ or 0.029 assuming duration of immunity following infection of approximately 18 years.

Through model calibration in the pre-vaccine era, we found the basic reproductive number (R0) of pertussis to be between 9.87 and 11.47 in Southern Ontario between 1880 and 1929. Assuming a 16% reporting rate of pertussis at the time and a duration of immunity of approximately 18 years, best-fit estimates yielded *β*_1_ = 11.334, *β_2_* = 0.050, *β_2_* = 0.029 (**Figure 2**).

**Figure 3:**
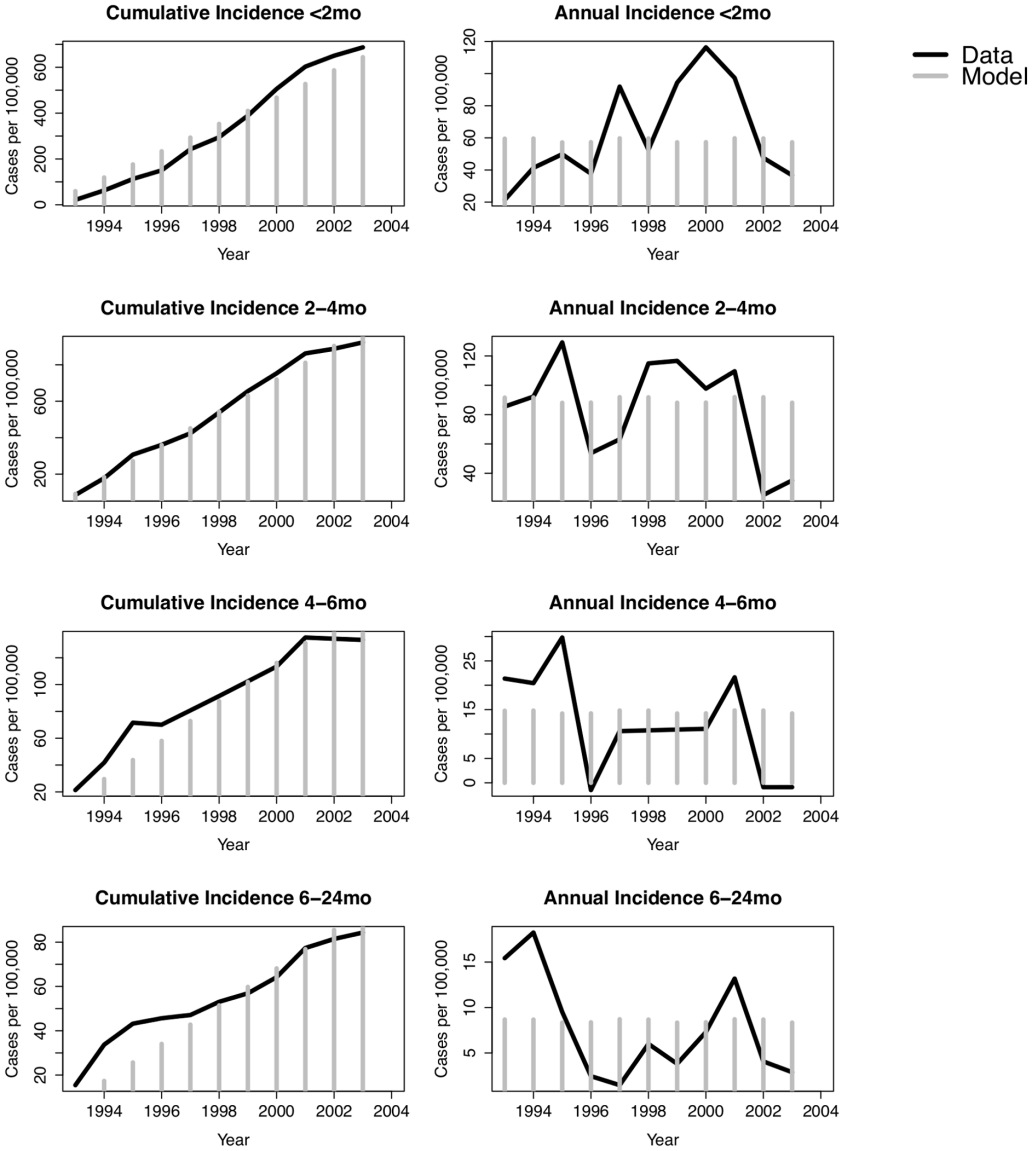
Model calibration to cumulative and annual pertussis incidence in the 1993–2004 vaccine era. Cases confirmed by the Public Health Laboratory —Toronto and the Hospital for Sick Children are shown in black and model predicted incidence of detected cases for the four age groups (under 2 months old, 2–4 months old, 4–6 months old, 6–24 months old) is shown in grey.

Using these best-fit estimates of natural history parameters, we used data from the vaccine era to derive estimates of duration of vaccine-induced immunity. The best-fit value was 5.42 years per vaccine dose received or 27.11 years for fully vaccinated individuals, assuming age specific case reporting rates. In particular, the best-fitting model had case-report values of 4.4%, 17.38%, 7.6%, and 0.55% for children under 2 months old, between 2 and 4 months old, between 4 and 6 months old, and between 6 months and 2 years old, respectively (**Figure 3**).

### Validation

The secondary outputs derived from the model were consistent with previously cited literature values. At 5.42 years per vaccine dose received, the estimated duration of vaccine-induced immunity was towards the shorter, rather than longer, end of previously reported ranges [11], [40], [41]. Our estimated reproduction number of 10.63 (seasonal range from 9.9 to 11.5), is a bit lower than previously cited reproduction numbers [42], [43]; however, this difference is likely due to heterogeneity between communities.

### Degree of Under-identification of Pertussis and Age-specific Effects

**Figure 4:**
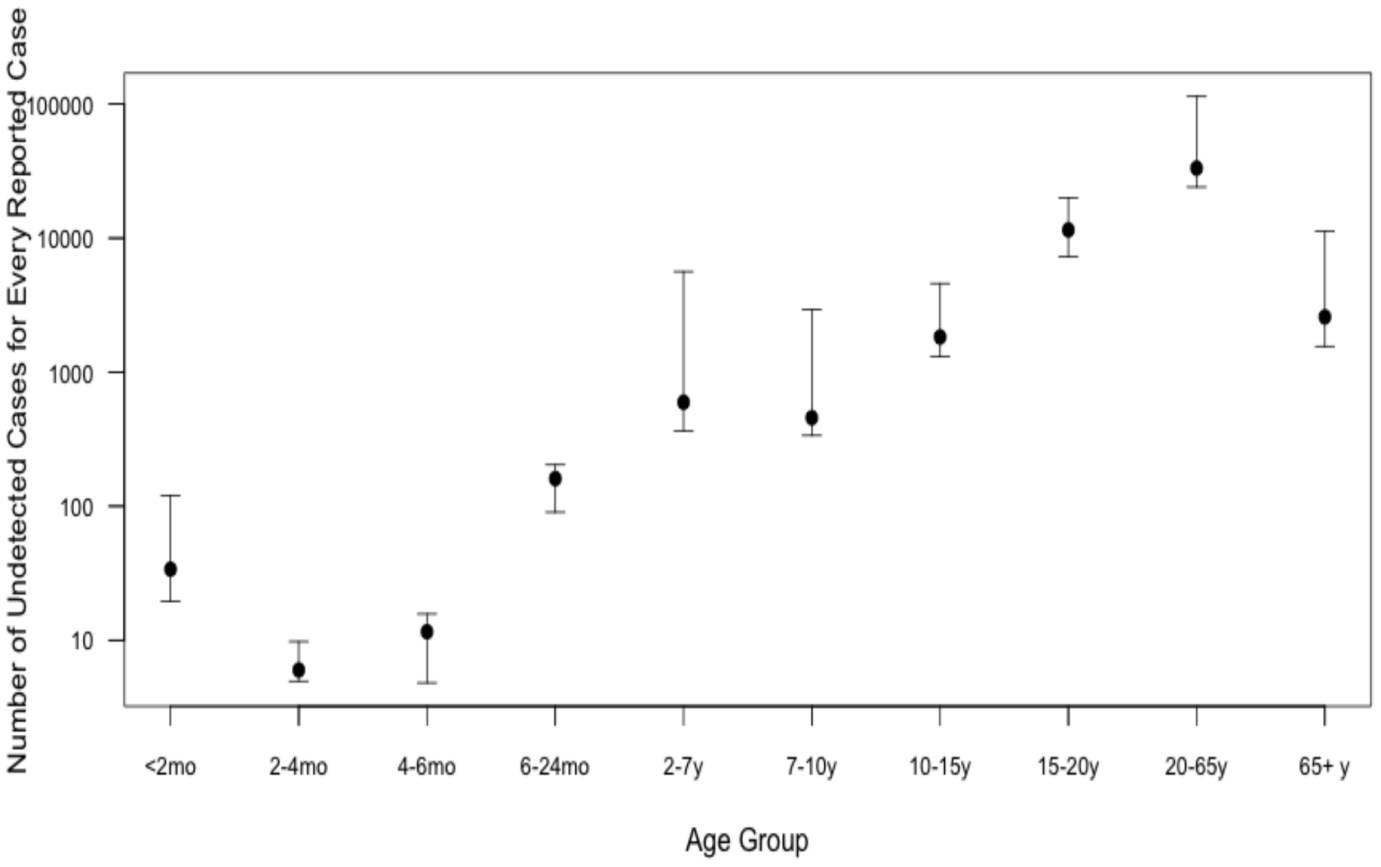
Estimated under-report of pertussis by age group. The model predicted ratio of underlying pertussis cases per laboratory-confirmed case was calculated based on reported cases for the Greater Toronto Area between 1993 and 2004. Results are presented on a logarithmic scale. For each age group, the minimum, mean, and maximum number of model predicted undetected cases for every reported case are displayed.

We used the model to estimate the annual age-specific incidence of pertussis and compared these rates to reported rates for the period between 1993 and 2004, to ascertain the likely degree of pertussis under-identification. Pertussis under-detection was found to vary dramatically with age: in the 2–7 year old age group, we projected that there were approximately 597 undetected pertussis cases per reported case, with this ratio increasing to a maximum of approximately 33,302 undetected cases per reported case in the 20 to 64 year age group (**Figure 4**).

We also assessed the contribution of infections in persons with loss of immunity to the rate at which susceptible individuals become infected (i.e., the force of infection). Using best-fit model parameters, we estimated that approximately 97% of infections in the <2 age group were attributable to infection from persons re-susceptible to infection through loss of naturally-acquired or vaccine-induced immunity. The high burden of disease caused by previously exposed individuals occurred despite our assumption that these individuals were one-fifth as infectious as individuals without prior immunity.

### Estimation of Vaccine-Induced Immunity Among Older Individuals

**Figure 5:**
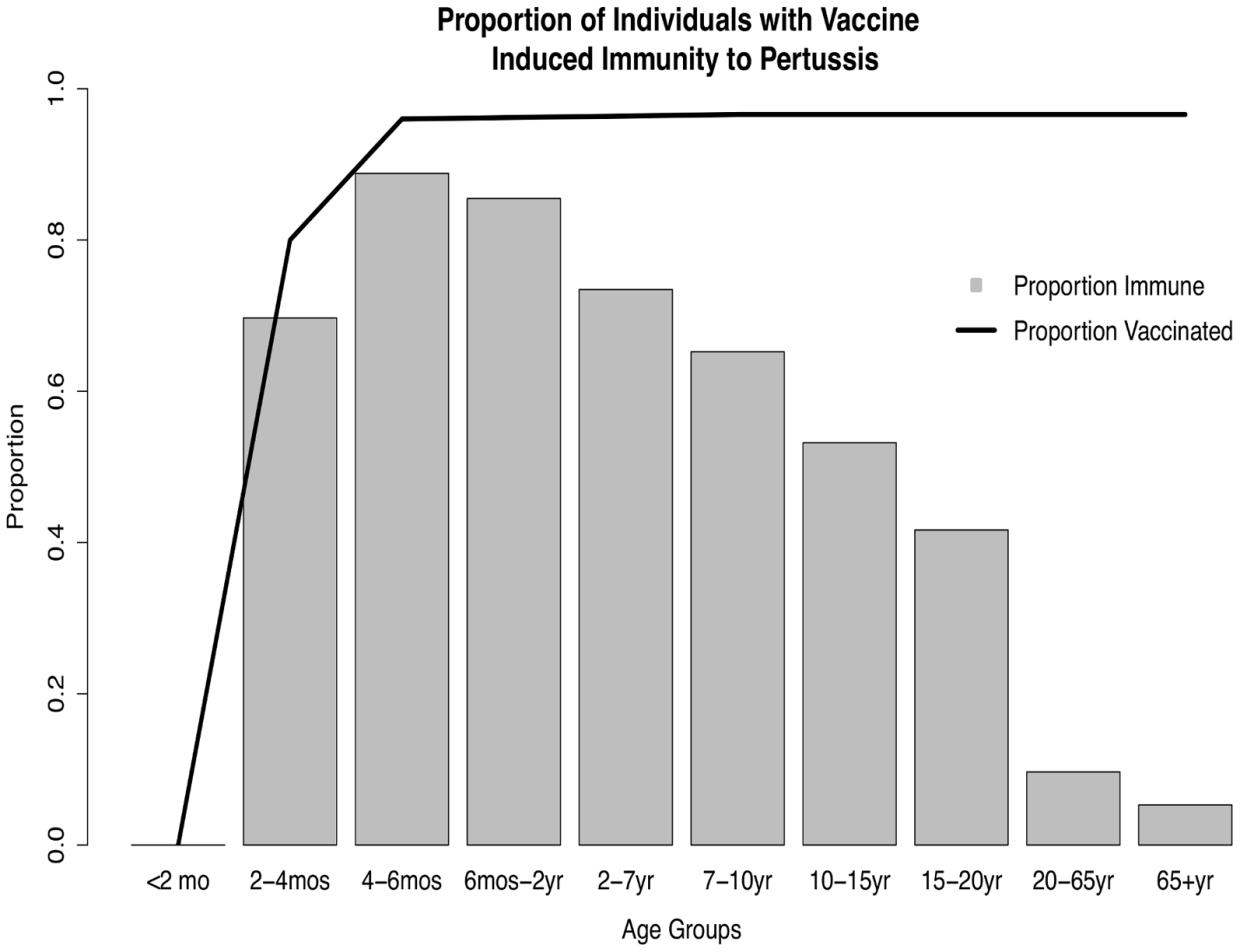
Model predicted vaccine induced immunity and vaccine coverage by age group, at end of 2012. Proportion immune represents the proportion of individuals who remain immune to pertussis due to vaccine-induced immunity. Proportion vaccinated represents the proportion of individuals who received at least one pertussis immunization.

We used the model predicted distribution of individuals in each of the vaccination compartments at the end of the year 2012 to ascertain the levels of vaccine-induced immunity in adolescents and adults of Southern Ontario (**Figure 5**). While we found high vaccination coverage rates among the older populations, less than 10% of individuals over age 20 were found to have immunity against pertussis. Infants and children were found to have the highest levels of immunity, with this value decreasing substantially in the older age groups.

### Impact of Uncertainty

**Figure 6:**
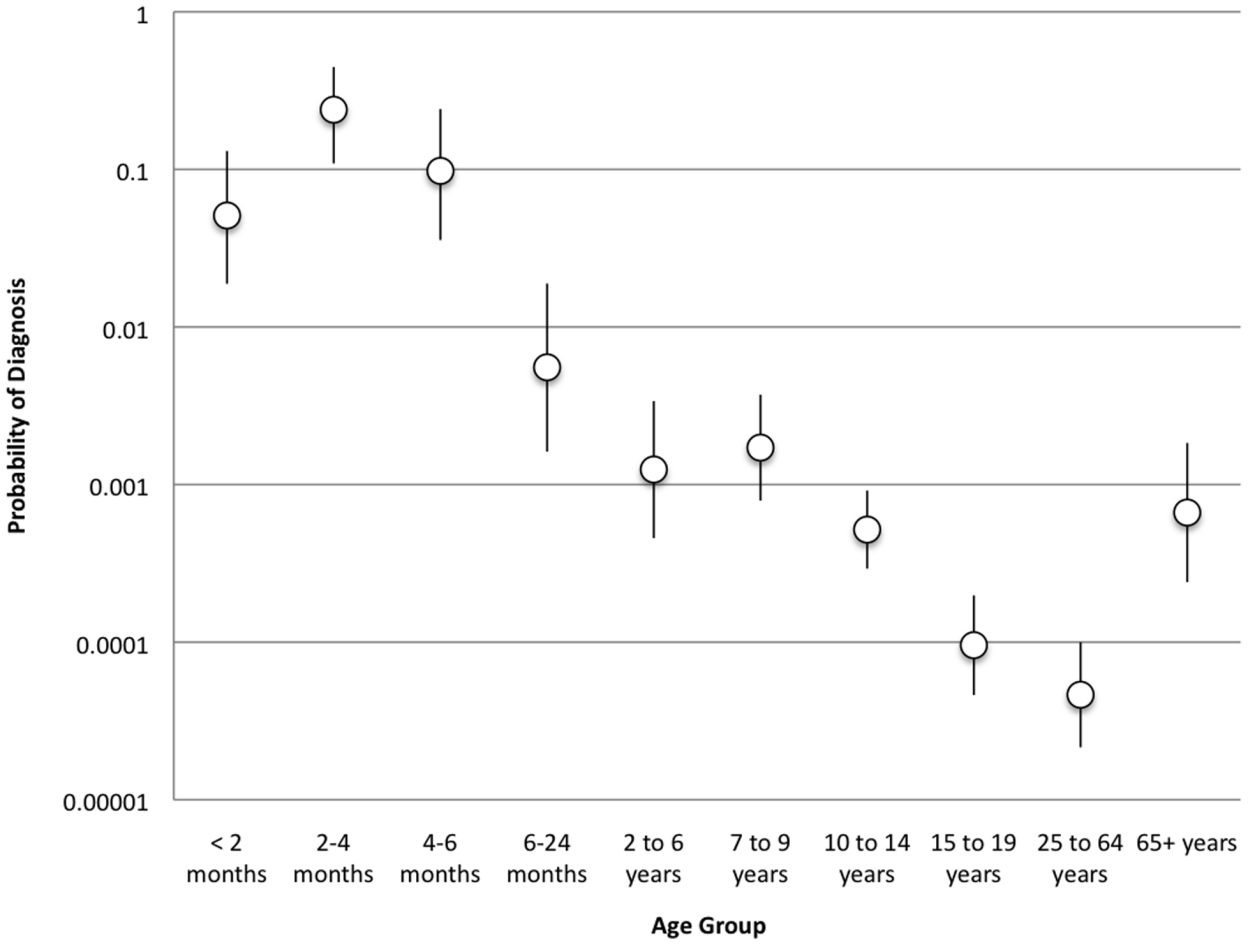
Multi-way sensitivity analysis. Probability of reported diagnosis for each age group. Confidence limits are based on the mean and standard deviation of the results from the sensitivity analysis.

Given the wide range of reported values for parameters describing the natural history of pertussis, we conducted wide-ranging sensitivity analyses to determine the impact of uncertainty on our estimates of pertussis under-identification. Results are presented in **Figure 6** **.** Although variation of input parameters across plausible ranges resulted in some variability in the estimated ratio of total pertussis cases to reported pertussis cases, there was no qualitative difference between results from sensitivity analyses and those derived in the base case.

## Discussion

The ongoing morbidity associated with pertussis even in the face of widespread pediatric immunization simultaneously highlights gaps in our knowledge regarding the epidemiology of this disease, and the importance of these very gaps. In both Canada and the United States, national immunization recommendations have evolved and now advocate boosting of adolescents and young adults against pertussis [27], [28], but whether such policy changes will result in further progress towards disease elimination remains to be seen. Some authors have expressed skepticism regarding this strategy, suggesting that the limited interactions between adolescents and infants and young children at a population level make it unlikely that interventions targeted at the former group would result in large reductions in risk in the latter [11]. A further element of complexity relates to the question of whether reduced force of infection resulting from immunization could paradoxically increase the pool of potentially infectable adolescents and adults by eliminating natural boosting [25].

The decrease in pertussis severity in older individuals (who presumably have partial immunity to the disease via prior exposure or vaccination) has been well described [44], [45] and a recent community-based study performed in Poland suggested that there is an age-related decrease in the likelihood that pertussis is reported to public health authorities [26]. This creates an obstacle to formulation of optimal disease control policy, as adults with mild disease are unlikely to modify their behavior in a way that prevents disease transmission, are less likely to present for clinical care and diagnostic testing, and as such are likely to be absent from surveillance records. We sought to create a mathematical model of pertussis in Southern Ontario, Canada to approximate the degree of under-reporting of pertussis in older individuals that would be expected based on reported pertussis epidemiology by age group.

The creation of well-calibrated disease dynamic models for pertussis is known to be challenging. By incorporating age-structure and limiting the duration of effective immunity following natural infection [33], we were able to calibrate our model such that it fit well to pre-vaccination time-series. Calibration of the model to reflect reported case counts in infants was greatly facilitated by assuming the existence of a partially immune state in which individuals with prior infection or vaccination were less infectious than individuals with first infection in the absence of prior immunity; this finding is similar to that previously reported by Broutin et al. [46], and consistent with the milder course of pertussis in older individuals [47]. Nonetheless, even assuming marked reductions in infectiousness in older individuals with pertussis these individuals are the source of most infections in our model.

In estimating the degree of under-identification of pertussis in older age groups, we projected that that age-related patterns of under-reporting are inverse to patterns of reported incidence, with pertussis under-reported by several orders of magnitude in adults. Again, this echoes the suggestion by others that pertussis is markedly under-reported in adults [26], [48], [49] and provides a credible explanation as to why high rates of vaccination coverage have failed to eliminate pertussis [6], [50].

Our model has important policy implications; in particular, that optimal control of pertussis may depend on repeated boosting of adults (as with diphtheria and tetanus). Our best-fit model also implies that the duration of protection through immunization is toward the shorter, rather than longer, end of reported ranges [40], [41]. With our estimated reproductive number of 10.63 and some seasonal variability in occurrence, we estimate the critical fraction vaccinated for herd immunity at 90–92%, which would be impossible in the absence of adult boosting. Fortunately, a safe combined vaccine preparation of diphtheria, tetanus, and pertussis, appropriate for use in adults, is now available, but the challenges associated with immunization of adults are well described [51]. Like any model, ours has limitations, and when data or parameters were sparse or absent we were forced to make some simplifying assumptions. However, our model outputs were robust over a wide range of possible parameter values, and in the face of alternate assumptions. As further data on the natural history and epidemiology of this disease become available, it will become possible to further refine the estimates presented here.

In conclusion, we were able to create a well-calibrated population dynamic model of pertussis that reproduced the reported epidemiology of this disease in children in Southern Ontario, Canada, both before the advent of immunization, and after immunization became widespread. Our model implies that maintenance of pertussis endemicity in the face of high rates of vaccine coverage depends on relatively short duration of immune protection from *both* natural infection and immunization, as well as continued susceptibility to infection (albeit with diminished infectiousness) in adults. While areas of uncertainty in our model suggest promising avenues for future research, our findings support the suggestion that ongoing pertussis boosting in adults may be necessary for optimal control of this disease in children.

## Supporting Information

File S1 (DOCX)
