# Supplementary material for: Estimation of the Underlying Burden of Pertussis in Adolescents and Adults in Southern Ontario, Canada
Source: PLoS One. 2013 Dec 23;8(12):e83850. doi: 10.1371/journal.pone.0083850 (PMC3871538; doi:10.1371/journal.pone.0083850)
Supplement: File S1 — (DOCX) [file pone.0083850.s001.docx]

**Model Description**

To model the transmission dynamics of pertussis in the presence of vaccination, we built an age-structured SEIR-type model that included heterogeneity in contact patterns by age. A schematic overview of the model is given in **Figure 1**, where *S*_j_(t), E_j_(t), I_j_(t), and R_j_(t) represent the respective number of susceptible, exposed, infectious, and recovered individuals in are groups j=1,2, …,10. SR_j_(t), ER_j_(t), and IR_j_(t) represent the number of susceptible, exposed, and infectious individuals who have been previously exposed to pertussis through infection or vaccination and whose immunity to infection has waned. Where applicable, V_j,k_(t) represents the number of individuals in each age group that have received k doses of vaccine with k=1,2,..,5. There are a total of five possible vaccinated compartments, representing receipt of between one and five total doses of vaccine. Vaccination was implemented according to the current immunization schedule, with a proportion of individuals receiving the vaccine as they enter an age category for which pertussis vaccination is recommended (i.e., as they enter the 2 month, 4 month, 6month, 2 years, or 7 years of age categories). The exposed and infected classes were divided into 2 and 4 compartments, respectively, to change the amount of time spent in each class from and exponential to a more realistic gamma distribution. We allowed the model to run for 145 years before implementation of vaccination.

The age groups are defined as follows:

1: 0-2 months

2: 2-4 months

3: 4-6 months

4: 6-24 months

5: 2-7 years

6: 7-10 years

7: 10-15 years

8: 15-20 years

9: 20-65 years

10: ≥65 years

**Model Equations**

$$\begin{matrix} \frac{dS_{j}}{dt}=\eta_{j}N-\lambda_{j}S_{j}+\left(1-c_{j-1,1}\right)\rho_{j-1}S_{j-1}-\rho_{j}S_{j}-\mu_{j}S_{j} \\ \frac{dV_{2..6,1}}{dt}=c_{j-1,1}\rho_{j-1}S_{j-1}+\left(1-c_{j-1,k+1}\right)\rho_{j-1}V_{j-1,1}+\omega_{v}V_{j,2}-\omega_{v}V_{j,1}-\rho_{j}V_{j,1}-u_{j}V_{j,1} \\ \frac{dV_{2..6,2..5}}{dt}=\left(1-c_{j-1,k+1}\right)\rho_{j-1}V_{j-1,k}+c_{j-1,k}\rho_{j-1}V_{j-1,k-1}+\omega_{v}V_{j,k+1}-\omega_{v}V_{j,k}-\rho_{j}V_{j,k}-\mu_{j}V_{j,k} \\ \frac{dV_{7..10,1..5}}{dt}=\rho_{j-1}V_{j-1,k}+\omega_{v}V_{j,k+1}-\omega_{v}V_{j,k}-\rho_{j}V_{j,k}-\mu_{j}V_{j,k} \\ \frac{dE_{j}}{dt}=\lambda_{j}S_{j}-\varepsilon E_{j}-\rho_{j}E_{j}+\rho_{j-1}E_{j-1}-\mu_{j}E_{j} \\ \frac{dI_{j}}{dt}=\varepsilon E_{j}-\gamma I_{j}-\rho_{j}I_{j}+\rho_{j-1}I_{j-1}-\mu_{j}I_{j} \\ \frac{dR_{j}}{dt}=\gamma\left(I_{j}+IR_{j}\right)-\omega R_{j}-\rho_{j}R_{j}+\rho_{j-1}R_{j-1}-\mu_{j}R_{j} \\ \frac{dSR_{j}}{dt}=-\lambda_{j}SR_{j}+\omega R_{j}+\omega_{v}V_{j,1}-\rho_{j}SR_{j}-\mu_{j}SR_{j} \\ \frac{dER_{j}}{dt}=\lambda_{j}SR_{j}-\varepsilon ER_{j}-\rho_{j}ER_{j}+\rho_{j-1}ER_{j-1}-\mu_{j}ER_{j} \\ \frac{dIR_{j}}{dt}=\varepsilon ER_{j}-\gamma IR_{j}-\rho_{j}IR_{j}+\rho_{j-1}IR_{j-1}-\mu_{j}IR_{j} \end{matrix}$$

**Model parameters**

| **Parameter** | **Description** |
| --- | --- |
| *λ__^*^* | Force of infection |
| *ε* | Rate of transition from exposed to infectious |
| *γ* | Rate of recovery from infection |
| *ω* | Rate of loss of immunity following infection |
| *ω_v_* | Rate of loss of immunity following vaccination |
| *rr* | Relative infectiousness of for individuals with previous exposure to pertussis |
| *c_j,k_* | Vaccine coverage |
| *ρ_j_* | Aging rate |
| *μ_j_* | Mortality rate |
| *η_j_* | Birth rate |

Subscript j indicates age group, k indicates vaccine dose number.

^*^Force of infection is given by:

$$\lambda_{j}=\sum_{m=1}^{10} \frac{\varphi_{jm}\beta(t)\left(I_{m}+rrIR_{m}\right)}{N}$$

where *φ_jm_* is the contact rate for infective individuals of age group m (*I_m_* and *IR_m_*) with susceptible individuals of age group j (based on a population-based prospective study of contact patterns in eight European countries), *rr* is the reduction in infectiousness for individuals with previous exposure to pertussis, *N* is the total population size, and *β(τ)* is the probability of transmission given contact (assumed to be independent of age):

$\beta(t)=\beta_{1}*\left(1+\beta_{2}\cos\left(\frac{2\pi t}{365}\right)+\beta_{3}\cos\left(\frac{2\pi t}{4*365}\right)\right)$.

**Model Calibration**

For calibration of the base model without vaccination, a time series of pertussis mortality in Ontario between 1880 and 1929 (1) was used to derive estimates of pertussis. Specifically, we used reported proportionate mortality by age group (1) and applied age-specific case-fatality ratios (estimated in 32 U.S. cities over a ten-year period) (2) to calculate expected pertussis incidence. In the absence of vaccination we assigned a duration of immunity after natural infection of approximately 18 years based on the best available data (3). We used an annual forcing term (*β_2_*), a seasonal forcing term (*β_3_*), and a base transmission parameter (*β_1_*) to encapsulate the underlying dynamics of the effective contact rate (*β*). *β_1_,* *β_2_*_,_ and *β_3_* were varied to achieve the optimal fit between model predicted incidence and the data for the under 2 years of age cohort. Pertussis is known to be under-diagnosed, and based on best available data we assumed a 16% probability of case-detection (4).

In order to calibrate estimates of duration of vaccine-induced immunity, we added vaccination to the best calibrated model derived based on natural history data as above. We then calibrated the model, incorporating vaccination, to a previously described time series including all data on laboratory-confirmed pertussis cases for the Greater Toronto Area (GTA), covering the period 1993 to 2004 (5). We varied age-specific case-report probabilities and duration of vaccine-induced immunity to achieve an optimal model fit to the data on individuals less than 2 years old. We used data on the under 2 age group for both model calibration steps because we assumed case-detection would be most complete for younger children, given more typical and more severe disease manifestations in this group.

**Contact Matrix**

Below is the contact matrix used in simulations, adapted from a prospective study of contact patterns in Great Britain (6). The values represent the average number of contacts in each column age group that an individual in each row age group meets per day. In this study, a ‘contact’ is defined as either a two-way conversation involving an exchange of at least 3 words (non-physical contact) or an interaction with skin-to-skin contact (physical contact).

|  | **0-2mo** | **2-4mo** | **4-6mo** | **6mo-2y** | **2-7y** | **7-10y** | **10-15y** | **15-20y** | **20-65y** | **65+ y** |
| --- | --- | --- | --- | --- | --- | --- | --- | --- | --- | --- |
| **0-2mos** | 0.002 | 0.002 | 0.002 | 0.019 | 0.047 | 0.013 | 0.014 | 0.008 | 0.130 | 0.013 |
| **2-4mos** | 0.002 | 0.002 | 0.002 | 0.019 | 0.047 | 0.013 | 0.014 | 0.008 | 0.130 | 0.013 |
| **4-6mos** | 0.002 | 0.002 | 0.002 | 0.019 | 0.047 | 0.013 | 0.014 | 0.008 | 0.130 | 0.013 |
| **6mos-2y** | 0.019 | 0.019 | 0.019 | 0.173 | 0.424 | 0.117 | 0.123 | 0.072 | 1.173 | 0.117 |
| **2-7y** | 0.051 | 0.051 | 0.051 | 0.460 | 2.138 | 1.828 | 0.682 | 0.436 | 4.658 | 0.506 |
| **7-10y** | 0.019 | 0.019 | 0.019 | 0.171 | 1.936 | 2.390 | 0.654 | 0.438 | 3.468 | 0.408 |
| **10-15y** | 0.016 | 0.016 | 0.016 | 0.144 | 0.812 | 0.786 | 6.850 | 1.520 | 4.540 | 0.740 |
| **15-20y** | 0.011 | 0.011 | 0.011 | 0.099 | 0.334 | 0.204 | 1.030 | 6.710 | 6.500 | 1.110 |
| **20-65y** | 0.166 | 0.166 | 0.166 | 1.491 | 5.138 | 3.234 | 5.090 | 5.950 | 70.490 | 10.330 |
| **65+ y** | 0.008 | 0.008 | 0.008 | 0.069 | 0.242 | 0.156 | 0.330 | 0.300 | 5.990 | 3.510 |

**References**

1. Ross MA. The mortality in Ontario of four communicable diseases of childhood. Can Public Health J. 1932:331-41.

2. Emerson H. Measles and whooping cough: incidence, fatality, and death rates in thirty-two cities of the United States, in relation to administrative procedures intended for their control - 1924-1933. Am J Public Health. 1937;27(6):Supplement.

3. Wendelboe AM, Van Rie A, Salmaso S, Englund JA. Duration of immunity against pertussis after natural infection or vaccination. Pediatr Infect Dis J. 2005 May;24(5 Suppl):S58-61.

4. Clarkson JA, Fine PE. The efficiency of measles and pertussis notification in England and Wales. Int J Epidemiol. 1985 Mar;14(1):153-68.

5. Fisman DN, Tang P, Hauck T, Richardson S, Drews SJ, Low DE, et al. Pertussis resurgence in Toronto, Canada: a population-based study including test-incidence feedback modeling. Bmc Public Health. 2011;11:694.

6. Mossong J, Hens N, Jit M, Beutels P, Auranen K, Mikolajczyk R, et al. Social contacts and mixing patterns relevant to the spread of infectious diseases. PLoS Med. 2008 Mar 25;5(3):e74.
